# The Associations of Built Environment with Older People Recreational Walking and Physical Activity in a Chinese Small-Scale City of Yiwu

**DOI:** 10.3390/ijerph18052699

**Published:** 2021-03-08

**Authors:** Jiabin Yu, Chen Yang, Xiaoguang Zhao, Zhexiao Zhou, Shen Zhang, Diankai Zhai, Jianshe Li

**Affiliations:** 1 Faculty of Sport Science, Research Academy of Grand Health, Ningbo University, Ningbo 315211, China; zhaoxiaoguang@nbu.edu.cn (X.Z.); zhouzhexiao@nbu.edu.cn (Z.Z.); nbuzhangshen@outlook.com (S.Z.); zdk5780245@outlook.com (D.Z.); lijianshe@nbu.edu.cn (J.L.); 2 Department of Kinesiology and Physical Education, McGill University, Montreal, QC H2W 1S4, Canada; chen.yang4@mail.mcgill.ca

**Keywords:** over 60 years old, leisure time, empirical study, correlates

## Abstract

Physical activity would bring in plenty of health benefits, especially recreational physical activity (RPA). Previous studies have suggested that built environment would affect older people’s recreational walking (RW) and RPA, but how the effects exist in a small-scale Chinese city remains unclear. Two hundred and fifty-two older participants were recruited in the city of Yiwu using cross-sectional survey of random samples in 2019. RW and RPA level of participants and perceived scores of built environments were collected using the International Physical Activity Questionnaire and Neighborhood Environment Walkability Scale, respectively. Linear regression analysis was conducted to investigate the association of built environment with older people’s RW and RPA. The results showed that two main factors affecting older people’s RW and RPA were residential density and aesthetics. Additionally, access to services was related to RW, and street connectivity was correlated with RPA. The associations of RW with built environment varied slightly with demographic variables included in the regression model. All the results suggested that lower residential density, better aesthetics environment, and higher street connectivity would motivate older people to engage more in RW and RPA. The better access to services encourages only RW, not RPA, in older people. These findings would be helpful for policy decision makers in the urban construction process in Yiwu. More studies are needed to enlarge the scientific evidence base about small-scale cities in China.

## 1. Introduction

Physical activity would bring in obvious and vast health benefits as plenty of previous papers have suggested [1,2]. The health benefits of physical activity include risk reductions in coronary heart disease, cancers, type 2 diabetes in physical health, and also improvements of depression, cognitive impairment, and social isolation in mental health [3,4,5]. There are four types of physical activity including work-related, family-related, traffic-related, and leisure-time physical activity [6]. Leisure-time physical activity would cause a higher health benefits due to greater energy consumption as World Health Organization (WHO) suggested [7]. Considering the worldwide population aging problem [8,9] and the prevalence of physical and mental diseases and functional losses with aging [10], taking part in physical activity is very important to keep a good health condition for older people. Especially, older people would experience greater health benefits from active physical activity than younger people [11]. Unfortunately, older people are usually inactive in the worldwide [3,12,13]. Therefore, increasing older people physical activity is vital for healthy aging and reducing economic burden of health care.

Motivators and barriers for older people physical activity include individual, social, and environment factors as previous studies suggested [14,15]. In the individual factors, some suggested motivators include health improvement and enjoying physical activity [15]. Barriers are poor health status, fear of injury and falling [16], and lack of time due to taking care of children [17]. Health status has been suggested to be both a motivator and barrier for older people in physical activity [18]. In social factors, social support was reported as a motivator. Providing information by health care providers and raising older people awareness about physical activity would motivate older people to begin exercise regimens [19]. In the environmental factors, some suggested motivators and barriers of older people include the supply of sports facilities, the availability of resting place, walking paths [20], and weather [21]. In general, a high level of older people physical activity could be observed in the spring, in sunny weather, and at moderate temperatures [15,22]. Meanwhile, environment should be considered from the broad perspectives of person, household, neighborhood, or community as the person–environment–occupation model suggested [23]. Additionally, with the coronavirus disease 2019 (COVID-19) pandemic worldwide, public places including parks and green space are closed, or open with limitations of visitor number and physical distance, in some countries. Undoubtably, this would be a barrier for older people physical activity. On the other hand, engaging in physical activity would decrease the damage caused by the COVID-19, and this might encourage older people to take part in more physical activities [24]. COVID-19 might fundamentally change the relationship of residents with public space, and affect the physical activity of residents [25,26,27]. According to the WHO report “Global Age-friendly Cities: A Guide”, the determinants of active aging include health and social services, behavioral determinants, personal determinants, physical environment, social determinants, and economic determinants [28]. All these factors being improved would motivate older people to be physically active and engage in more physical activity. 

Numerous previous studies have indicated that built environment plays an important role in older people physical activity, and some of them especially focused on older people’s RW and RPA [29,30,31,32,33,34,35]. In a study with 13,745 older participants from 12 countries, Sugiyama et al. observed that older people’s RW was linearly associated with perceived residential density, land use mix, street connectivity, aesthetics, safety from crime, and proximity to parks [34]. In Hong Kong, Cerin et al. found that older Chinese people reported two to four times the level of RW compared to older Western people, but the older people’s RW in Hong Kong showed less association with the built environment [33]. For older people’s RPA, Van et al. suggested that older people’s overall RPA was positively associated with access to recreational facilities and parks/open space, in a review study including 72 papers. They also found that older people’s RW was positively related to walkability, land use mix access, and aesthetically pleasing scenery [35]. Therefore, the associations of older people’s RW and RPA with the built environment would vary to some extent, and it is essential to investigate built environment motivators and barriers of older people’s RW and RPA. 

There are a few studies investigating the effect of built environment on Chinese adults [29,30,31,32,33,36,37,38,39,40,41,42], and some of them focused on older people [29,30,31,32,33,36,37,38]. However, considering the huge number of population and the diversity of the city environment in China, more studies are still needed. The associations between the built environment and older Chinese people’s physical activity in previous studies varied mainly because the participants’ demographic characteristics and social-economic status were different in diverse-scale cities. For example, in a comparative study of two cities, Yu et al. found that residential density was only related to older people’s RPA in Wenzhou, and access to services was only related to older people’s RPA in Hangzhou [27]. To the best if our knowledge, all previous Chinese studies targeted large-scale and mid-scale cities, and none of them surveyed the small-scale city. How the built environment affects older Chinese people’s physical activity in a small-scale city was still unknown. Therefore, the purpose of this study was to investigate the associations of the built environment with older people’s RW and RPA in a small-scale city of Yiwu. Additionally, we would like to confirm whether the associations would differ between older people’s RW and RPA. The hypothesis of this study was that the association of the built environment with older people’s RW and RPA would be different to some extent. The finding of this study would broaden the Chinese scientific database in this field and could provide suggestions of urban construction for policy decision maker in Yiwu. 

## 2. Materials and Methods

### 2.1. Sample and Study Design

This study was conducted in the city of Yiwu, which is a small-scale city of Zhejiang province in the eastern region of China. Compared with the cities surveyed by previous Chinese studies [29,30,31,32,33,36,37,38,39,40,41,42], Yiwu is smaller both in the resident population and economic development. The official population was 810,000 in 2019. The city of Yiwu is famous for small commodity wholesale, and has been awarded with the largest small commodity entrepot by the United Nations and the World Bank. Yiwu wholesale market is granted to be the first 4A level shopping and tourism place by China national tourism administration. In the dwelling environment, Yiwu has been awarded with national titles of sanitary city, environment protection model city, garden city. For public transportation, the main traffic mode is bus for the residents in Yiwu now. Two subway lines are under construction and will be opened at the end of 2021.

The surveyed area of this study was Choucheng, which is the core downtown area of Yiwu based on geographical location. A cross-sectional survey of random samples was carried out by three team members from July to December in 2019. Some efforts to reduce potential sources of bias are as follows. (a) Eight different communities were included in this study so that the bias of using the same source can be reduced. Older participants were recruited with the help of community resident committees. (b) All team members were trained before the survey in order to be familiar with the data collection process and understand the surveyed questions precisely. One-to-one interviews were conducted to ensure the data quality and avoid participants’ bias as much as possible. (c) The inclusion criteria of participants of this study were older people over the age of 60 years old, residents of the selected communities and have lived in the community for at least 6 months, and older participants with normal communication ability and without cognitive impairment. The older people who did not satisfy the above criteria were excluded from the survey. In the one-to-one interview, the uncompleted questionnaires were deleted and were not counted. Eventually, the 252 participants who completed all the questions were included in this study. The sample size in this study is comparable with previous relative studies [29,31].

### 2.2. Measures

The basic demographic data were collected using an individual characteristic questionnaire including age, gender, education level, income situation, travel mode choice, and motion sickness. The levels of RW and RPA were collected using the International Physical Activity Questionnaire-Short version (IPAQ-S). IPAQ-S evaluated the level of walking, moderate physical activity, and vigorous physical activity. The reliability and validity of IPAQ-S have been testified by a previous review study [43]. Physical activity source data were then converted into Metabolic equivalent scores (MET) based on the IPAQ scoring procedure. The MET value of walking represented RW level. The summed MET value of walking, moderate physical activity, and vigorous physical activity represented RPA level. 

The built environment was evaluated using the Chinese version of the Neighborhood Environment Walkability Scale-Abbreviated (NEWS-A). The evaluated built environments include the participants’ communities and the neighborhood with 10–15 min walking distance. The reliability and validity of NEWS-A have been confirmed by a previous study [44] and has been widely used in previous studies [29,30,32,33,40]. The detailed description of NEWS-A could be found in our previous study [30]. Briefly, eight built environment elements were evaluated by this questionnaire. Twenty questions of land use mix diversity evaluated distances from home to destinations. A higher score means a farther distance. The five questions of residential density were on a 5-point Likert Scale (1—none, 5—all of them). A larger score indicates a higher residential density. Twenty-six questions of the other six built environment elements were on a 4-point Likert Scale (1—totally disagree, 4—totally agree). A higher score means a better perceived built environment. The average score of each built environment element represents its final score.

### 2.3. Statistical Analysis

The descriptive statistics of frequency and percentage were used to describe participants demographic characteristics. Means and standard deviations were used to describe the average score of each built environment element and the average level of RW and RPA in all participants. A multivariate linear regression method with a univariate was conducted to investigate the association between built environment and older people’s RA and RPA. Model 1 is the regression analysis without demographic variables as covariables. Model 2 is the regression analysis with demographic variables as covariables. The statistically significant level was set at *p* < 0.05. SPSS 19.0 software was used to conduct all the analyses (SPSS Inc., Chicago, IL, USA).

## 3. Results

Table 1 shows the demographic characteristics of older people in the city of Yiwu. Most (82.6%) participants in this study were 60–79 years old, and only 17.4% of participants were over 80 years old. The education level of 86.2% older people in this study was lower secondary or below. In the income situation, 38.1% of older people in this study had 1501–2500 RMB every month. The percentages of the other four income levels were quite similar. Most participants chose car or bus as their travel mode. The percentage of choosing walking was slightly higher than that of choosing bicycle. 

Table 2 shows the perceived scores of eight built environment elements and the total scores of RW and RPA. Except land use mix diversity that evaluated the distances from home to destinations, three other built environment elements scores averagely reached about 3 or above including access to services, street connectivity, and walking/cycling facilities. These results suggested that older people in this study were partly satisfied with the three built environment elements. Three built environment elements that averagely scored lower than 3 were aesthetics, pedestrian/traffic safety, and crime safety, which meant that the participants’ attitude towards the three built environment elements were between partly dissatisfaction and partly satisfaction. Regarding participant physical activity, the score of RW was about 300 MET min/week lower than that of RPA. RW was the main method of RPA when older people went out to take exercise in this study. 

Table 3 shows the association of built environment with older people’s RW and RPA. Residential density and aesthetics were found to be significantly associated with both RW and RPA. Residential density was negatively related to participants RW and RPA, while aesthetics was positively associated with participants RW and RPA. Additionally, access to services was only positively associated with participants RW. Street connectivity was only positively related to participants RPA significantly. No significant associations with RW and RPA were found in other built environment elements. 

Table 4 shows the association of built environment with older people’s RW and RPA with demographic variables as covariables in the model. One more built environment element was found to be significantly related with RW compared with the results in Table 3. Street connectivity was positively associated with participants RW. The association between built environment elements and participants RPA did not change compared with the results in Table 3. For demographic variables, education level and income situation were found to be significantly associated with both RW and RPA. Education level was positively related with participants RW and RPA, and income situation was negatively associated with participants RW and RPA. No significant association with RW and RPA were found in the other built environment elements and demographic variables.

## 4. Discussion

The purpose of this study was to locate the association of perceived neighborhood-built environment with older people’s RW and RPA in a small-scale city of Yiwu in the eastern region of China. Especially, we endeavored to find whether the association would differ between RW and RPA. The finding of this study could be helpful for quantifying the effect of the built environment on older people’s RW and RPA and could provide some suggestions of urban construction for policy decision maker. 

### 4.1. RW as The Most Popular RPA in Older People

Our results showed that RW is the main way that older people engage in RPA, and only a small part of older people would choose to take part in moderate to vigorous intensity physical activity. The total score of RPA was 300 MET min/week, slightly higher than that of RW. Two hundred and fifty-two participants self-reported RW while only 52 of them reported moderate to vigorous intensity physical activity. All of the above results suggested that RW is the first and the most favorable choice when older people engage in outdoor physical activity in their leisure time. WHO suggested that older people should take part in moderate intensity physical activity for at least 150 min every week [7]. Although the benefits from moderate intensity physical activity are obvious [3], the percentage of older people who achieve the recommendation of physical activity level decreases with growing age. The results of global health survey published by WHO in 2015 found that about one-third of people aged between 70 and 79 years and one-half of people aged over 80 years do not reach the recommended physical activity level [45]. The situation is more serious in the city of Yiwu because only one-fifth of older people self-reported moderate to vigorous physical activity. The reasons for the low participation in moderate to vigorous physical activity might be barriers like poor health status and fear of falling as previous studies suggested [18,46,47]. Hence, how to build an age-friendly neighborhood environment in the urban construction process to help older people overcome fears like falling is a vital question needed to be considered for policy decision makers. Unfortunately, although some Chinese studies investigated the effect of built environment on older people’s physical activity [29,30,31,36,37,38], none of them especially focused on the influence of the built environment on older people’s moderate to vigorous intensity physical activity. Due to the small sample size in our study, we also failed to locate this association. Therefore, more studies are needed to investigate the question in China to provide scientific data for policy decision makers. 

### 4.2. The Association Similarities and Differences between RW and RPA

In this study, the associations of the built environment with older people’s RW and RPA were quite similar. This is mainly caused by the fact that only one-fifth of participants self-reported moderate to vigorous physical activity, which means that the data of four-fifths of the participants were the absolute same in RW and RPA. Our results showed that residential density and aesthetics were common factors in older people’s RW and RPA no matter which model was used. For demographic variables, educational level and income situation were common factors in older people’s RW and RPA. However, some differences were found in the associations of built environment with older people’s RW and RPA. Access to services was positively related to older people’s RW, but no significant association between older people’s RPA and access to services was found independent of the model used. This result might suggest that access to services is an important consideration when older people take part in walking in their leisure time. However, when moderate to vigorous physical activity is considered in RPA, access to services becomes less important for older people engaging in RPA. Additionally, street connectivity is only positively associated with older people’s RPA without demographic variables as a covariable in the model. This result might indirectly suggest that street connectivity is an important factor to encourage older people to take part in moderate to vigorous physical activity. 

### 4.3. Built Environment Factors Impacting RW and RPA in Older People

Residential density was negatively associated with older people’s RW and RPA, which means that a lower residential density would motivate older people to engage in RW and RPA in the small-scale city of Yiwu. This finding is in line with few Chinese studies [39]. Sun et al. found that a lower residential density would motivate women adults to spend more time in RW. However, the finding of this study was in conflict with numerous previous studies [34,36,48,49,50,51,52]. Besides, previous studies from our team [29,30] also observed a positive association between residential density and RPA. Yu et al. found that older people’s RPA was significantly positively associated with residential density in the mid-scale city of Jinhua, and they suggested that a higher residential density would be helpful for older people to find companions when they take part in outdoor activities like square dancing, which is very popular in China [29]. The association differences might be caused by diverse demographic characteristics and social-economic status of participants between this study and previous studies. The differences in those characteristics would absolutely have an effect on the association between built environment and older people’s RPA. To our knowledge, all the surveyed cities in previous Chinese studies were super large-scale, large-scale, or middle-scale cities. None of them focused on the small-scale city like Yiwu in this study. Whether the negative association between residential density and older people’s RPA would also exist in other small-scale Chinese cities needs more future studies. Additionally, walking behaviors would vary between different population density cities. In a systematic review, Yun et al. suggested that in densely populated cities like Hong Kong, older people walked mostly for both transport and recreation, but in lo-density cities, American older people walked less for transport and more for recreation [53]. 

This result indicated that a pleasing aesthetics environment would motivate older people to engage in more RW and RPA. In NEWS-A used in this study, aesthetics assessed green space, air cleanliness, cultural landscape, and attractive buildings. A higher aesthetics score means a better perceived environment related to these elements. This finding is consistent with plentiful previous studies [30,32,34,40,47,54,55,56]. Gong et al. found that older men living in neighborhoods with more green space participate more in regular physical activity compared with those living in neighborhoods with less green space [56]. In a review study, Barnett et al. also suggested that strong evidence supported the positive effect of aesthetically pleasing scenery on older people’s levels of total physical activity and walking, but the positive effect was limited in the studies of perceived measures used. They also suggested that as well as increasing older people physical activity, the benefits of good aesthetics also include reducing urban heat island effect, air pollution, and global disease burden [47]. In our opinion, a pleasing aesthetic environment would be beneficial in making older people feel relaxed and maintaining a good mood when they participate in outdoor activities, and eventually motivate them to engage more in physical activity. Except the perceived evaluation of aesthetics, aesthetics could be also evaluated by objective method like contemplative landscape model (CLM) as previous study suggested. CLM is useful for distinguishing and assessing landscape patterns and providing suggestions for urban greening strategy [57]. Therefore, how to build a pleasing aesthetics environment to increase older people’s RA and RPA is a vital consideration for policy decision makers in China, especially for the small-scale city as the results suggested in this study. 

Access to services was positively related to RW. However, with moderate to vigorous physical activity counted, the significant association disappeared between access to services and RPA. This result might indicate that access to services is an important consideration when older people take part in walking in their leisure time, but it is not a vital consideration for older people engaging in moderate to vigorous physical activity. The possible explanation of this result might be that the perceived score of land use mix diversity in this study was 3.02, which means that older people almost need 11 to 15 min walking from home to destinations. Such distance is friendly enough for older people walking as previous study suggested [29]. A good access to services would provide older people pleasant convenience to public places like commercial facilities and public transportation stations and would motivate older people to choose walking to these destinations instead of choosing other travel modes. Therefore, a positive association between access to service and older people’s RW was found in this study. The similar results were also found in previous studies [47,48,53,58]. Boakye et al. found that older people in Hong Kong reported more minutes of walking and a higher accessibility to destinations than their Brisbane counterparts, and the team suggested that good access to services would motivate older people to engage in more walking [48]. 

Street connectivity was positively associated with older people’s RPA independent of the model used, and positively associated with older people’s RW with demographic variables as covariables. The results suggested that a good street connectivity would motivate older people to engage more in RW and RPA. This finding is consistent with previous studies [31,47,49,58,59]. In a recently published umbrella review study, Bonaccorsi et al. showed strong evidence supporting the idea that street connectivity was positively associated with older people’s physical activity [49]. In demographic variables, educational level was found to be positively related to older people’s RW and RPA, which means that older people with a higher educational level would take part in more RW and RPA. Additionally, income situation was found to be negatively related to older people’s RW and RPA. This finding is in line with our previous study [29], but inconsistent with another Chinese study [31]. Wu et al. suggested that older adults with a high income were more likely to take part in RPA in the city of Nanjing [31]. The association differences between our studies and Wu et al.’s study might be due to the surveyed city scale differences. The targeted cities in our studies were mid-scale and small-scale cities, but Wu et al.’s study focused on a large-scale city. Whether the differences in the association between older people’s RPA and income situation would also exist in other Chinese cities needs more future studies. 

### 4.4. Strength and Limitation of This Study

The strength of this study is that we investigated the association of the built environment with older people’s RW and RPA at the same time. This is helpful for us to successfully locate the common built environment factors and different factors of older people’s RW and RPA. As we found, RW and RPA were affected by quite similar built environment elements because RW is the first and most common way that older people engage in RPA. Nevertheless, different built environment factors contributing to RW and RPA were also found, like access to services, which was only significantly related to RW. Secondly, considering the large population and national territory area, the number of Chinese studies focusing on the association of built environment and older people’s physical activity was few in number and needed to be enlarged more. Especially, most of the previous Chinese studies targeted cities of above mid-scale, and none of them surveyed small-scale cities. This study filled the gap and enlarged the scientific evidence base in this field. The limitation of this study is as follows. Firstly, due to self-reported tools used in this study, a few source biases were inevitably included into the data, and might bring an effect on the association of built environment and older people’s RW and RPA. Objective tools used might reduce the source bias to some extent as previous study suggested [37], but they also have some limitations like ignoring participant’s subjective feeling more or less. Secondly, only demographic variables were combined into linear regression analysis. Other variables like social and psychological variables that were suggested to be related with older people physical activity were not included. Thirdly, the results of this paper should be extended to other populations and countries with great caution because of different cultures and social-economic status of participants. The results of this study were only based on the current sample size in the city of Yiwu. Whether the association between older people’s physical activity and the built environment would vary with a larger sample size and how the association would be in other small-scale Chinese cities needs further study. 

## 5. Conclusions

RW is the main way that older people take part in RPA. The lower residential density, higher aesthetic environment, and higher street connectivity would motivate older people to engage in more RW and RPA in the city of Yiwu. Good access to service would encourage older people to take part in more RW. Moreover, older people with higher educational level and lower income are more likely to engage in RW and RPA. 

The results of this study should be considered by policy decision makers in the urban construction process in the city of Yiwu. In the current situation, older people always choose walking in their leisure time physical activity, so the built environment factors of older people’s RW including residential density, aesthetics, street connectivity, and access to services should be considered first. Considering the vast benefits that older people could obtain from moderate to vigorous physical activity, the built environment factors of this type of physical activity should also be considered in order to encourage older people to take part in more moderate to vigorous physical activity. To the best of our knowledge, none of Chinese published papers focused on this topic to support the policy decision. Hence, relative studies are called for in the future. 

## Figures and Tables

**Table 1:** Demographics of the participants in the city of Yiwu (*n* = 252).

Demographics Variable	*n*	*%*
Gender		
Men	120	47.6
Women	132	52.4
Age		
60–69 years	132	52.4
70–79 years	76	30.2
≥80 years	44	17.4
Education level		
Primary or below	100	39.7
Lower secondary	92	36.5
Upper secondary	32	12.7
Tertiary and above	28	11.1
Income situation (RMB) *		
≤1500	36	14.3
1501–2500	96	38.1
2501–3500	40	15.9
3501–4500	36	14.3
≥4501	44	17.4
Travel mode choice		
Car or bus	188	74.6
Bicycle	16	6.3
Walking	48	19.1
Motion sickness		
Yes	120	47.6
No	132	52.4

Note: * Income situation indicates the monthly income of the participant. RMB stand for Chinese currency Ren min bi, and the unit of RMB is yuan. % represents the percentage of each variable in the total participants in Yiwu.

**Table 2:** The perceived built environment scores, recreational walking (RW), and recreational physical activity (RPA) level of older people in the city of Yiwu (*n* = 252).

Variable	Score	Score Ranges	95% CI
Residential density	559.62 ± 62.63	413–738	(551.89, 567.35)
Access to services	2.99 ± 0.34	1.83–4.00	(2.95, 3.03)
Street connectivity	3.11 ± 0.45	1.67–4.00	(3.05, 3.17)
Walking/cycling facilities	3.06 ± 0.27	2.50–3.83	(3.03, 3.09)
Aesthetics	2.72 ± 0.37	1.80–3.60	(2.67, 2.77)
Pedestrian/traffic safety	2.33 ± 0.58	0.67–3.00	(2.26, 2.40)
Crime safety	2.43 ± 0.63	1.00–3.00	(2.35, 2.51)
Land use mix diversity	3.02 ± 0.35	2.25–4.10	(2.98, 3.06)
RW	1656.29 ± 1076.28	99–5544	(1523.40, 1789.18)
RPA	1955.33 ± 1312.05	198–5838	(1793.33, 2117.33)

Note: The units of RW and RPA are MET. min/week. 95% CI indicates the 95% confidence interval values.

**Table 3:** Association of built environment with older people’s RW and RPA in the city of Yiwu.

Built Environment Element		RW			RPA	
	*B*	*SE*	*p*	*B*	*SE*	*p*
Residential density	−3.05	1.00	0.003 *	−3.41	1.17	0.004 *
Access to services	340.70	169.91	0.046 *	237.17	198.19	0.23
Street connectivity	286.78	163.65	0.08	822.57	190.90	<0.001 *
Walking/cycling facilities	−26.91	217.58	0.90	−441.17	253.81	0.08
Aesthetics	776.46	189.57	<0.001 *	1080.267	221.12	<0.001 *
Pedestrian/traffic safety	−57.60	143.66	0.69	178.54	167.58	0.29
Crime safety	−101.91	127.18	0.42	−201.13	148.36	0.18
Land use mix diversity	−63.41	170.46	0.71	−300.21	198.84	0.13

Note: Depend variable: total score of older people’s RW or RPA, B stands for regression coefficient, SE represents standard error, * indicates significant difference (*p* < 0.05). Model fit of RW: R^2^ = 0.74, F = 86.21, *p* < 0.001. Model fit of RPA: R^2^ = 0.75, F = 91.39, *p* < 0.001.

**Table 4:** Association of built environment with older people’s RW and RPA when demographic variables as a covariable in the city of Yiwu.

Variable		RW			RPA	
	*B*	*SE*	*p*	*B*	*SE*	*p*
Residential density	−2.89	0.99	0.004 *	−3.13	1.15	0.007 *
Access to services	409.88	171.11	0.017 *	331.18	199.68	0.10
Street connectivity	379.53	165.99	0.023 *	864.37	193.71	<0.001 *
Walking/cycling facilities	19.53	237.95	0.94	−361.17	277.68	0.20
Aesthetics	799.81	183.05	<0.001 *	1098.02	213.61	<0.001 *
Pedestrian/traffic safety	−175.52	140.72	0.21	67.67	164.21	0.68
Crime safety	−69.75	125.16	0.58	−209.93	146.06	0.15
Land use mix diversity	−172.16	184.80	0.35	−393.07	215.66	0.07
Gender	−72.97	164.71	0.66	156.45	192.21	0.42
Age	−83.94	109.52	0.44	−247.38	127.81	0.06
Education level	305.28	79.16	<0.001 *	269.83	92.38	0.004 *
Income situation	−274.71	65.31	<0.001 *	−200.28	76.21	0.009 *
Travel mode choice	10.75	94.42	0.91	116.60	110.20	0.29
Motion sickness	93.58	135.65	0.49	−178.16	158.31	0.26

Note: Depend variable: total score of older people’s RW or RPA, B stands for regression coefficient, SE represents standard error, * indicates significant difference (*p* < 0.05). Model fit of RW: R^2^ = 0.77, F = 56.15, *p* < 0.001. Model fit of RPA: R^2^ = 0.78, F = 59.34, *p* < 0.001.

## Data Availability

Data available on request due to restrictions privacy. The data presented in this study may be available on request from the corresponding author and with authorization of funding origination.
